# Clinical, hormonal, and treatment outcomes in 247 patients with acromegaly: a single tertiary center experience

**DOI:** 10.55730/1300-0144.6205

**Published:** 2026-03-17

**Authors:** Ümran GÜL, Bekir UÇAN, Hayri BOSTAN, Hakan DÜĞER, Hayri KERTMEN, Caner ÜNLÜER, Halil DURANTAŞ, Oğulcan BOZ, Ceren KARAÇALIK ÜNVER, Burak MENEKŞE, Sema HEPŞEN, İlknur ÖZTÜRK ÜNSAL, Mustafa ÖZBEK, Erman ÇAKAL, Muhammed KIZILGÜL

**Affiliations:** 1 Division of Endocrinology and Metabolism, Department of Internal Medicine, Etlik City Hospital, Ankara, Turkiye; 2 Division of Endocrinology and Metabolism, Department of Internal Medicine, Çanakkale Mehmet Akif Ersoy State Hospital, Çanakkale, Turkiye; 3 Division of Endocrinology and Metabolism, Department of Internal Medicine, Medical Park Hospital, Antalya, Turkiye; 4 Department of Neurosurgery, Etlik City Hospital, Ankara, Turkiye

**Keywords:** Acromegaly, GH, IGF-1, treatment, long-term follow-up

## Abstract

**Background/aim:**

This study aimed to evaluate the demographic, clinical, and hormonal characteristics; comorbidities; surgical pathology; treatment modalities; and long-term clinical outcomes of patients diagnosed with and managed for acromegaly at our tertiary referral center over a 15-year period.

**Materials and methods:**

Between 2010 and 2024, 247 patients with acromegaly were evaluated. Diagnosis and biochemical remission were determined according to the 2024 Acromegaly Consensus Criteria.

**Results:**

The median follow-up duration was 8 years (IQR: 3–14). At the time of diagnosis, the most frequent pituitary hormone deficiency was secondary adrenal insufficiency, present in 17.8% of patients. The most common comorbidities were hypertension (HT, 42.1%) and diabetes mellitus (DM, 30.8%). At the time of diagnosis, macroadenomas were present in 153 of 193 patients (79.3%), and, according to the Knosp classification, 28.6% of tumors were grade 4. A total of 218 patients underwent surgical intervention; 86 (39.4%) achieved postoperative cure without the need for adjuvant medical therapy or radiotherapy. Postoperatively, one or more anterior pituitary hormone deficiencies were found in 34 patients (22.8%), with central hypothyroidism being the most common (52.9%). Patients who achieved biochemical remission were significantly older (p = 0.003), had a higher prevalence of HT and DM (both p < 0.05), and were more likely to exhibit positive growth hormone (GH) immunostaining (p = 0.017). In contrast, those who did not achieve remission were more likely to have higher Knosp grades (3–4) (p = 0.002).

**Conclusions:**

In this study, a favorable prognosis was associated with older age at diagnosis, lower baseline GH levels, positive GH immunostaining, smaller tumor size, and lower Knosp grade, underscoring the prognostic value of both biochemical and anatomical parameters. Further studies are warranted to refine prognostic markers and establish standardized treatment algorithms to improve long-term outcomes in acromegaly.

## 1. Introduction

Acromegaly is a rare endocrine disorder caused by excessive secretion of growth hormone (GH) and its peripheral target hormone, insulin-like growth factor 1 (IGF-1), with a reported prevalence of 2.8–13.7 cases per 100,000 individuals [1]. Acromegaly is primarily caused by hypersecretion of GH from benign pituitary adenomas and rarely by secretion of ectopic GH-releasing hormone [2]. Acromegaly is an insidious condition, often marked by a prolonged delay between the onset of symptoms and the eventual diagnosis [3]. Most patients are diagnosed in mid-adulthood, typically in the fifth decade of life. Large epidemiological studies and metaanalyses report mean ages at diagnosis ranging from the fourth to the fifth decade, with some cohorts showing slightly higher mean ages in women than in men, although substantial heterogeneity exists across populations. Increasingly, acromegaly is also being diagnosed in patients older than 65 years, whereas pediatric and young adult cases remain rare [4, 5]. The clinical features of acromegaly result from the persistent over-secretion of GH, which stimulates hepatic production of IGF-1, and from the mass effect of the pituitary tumor [6]. In addition to morphological changes such as acral overgrowth, soft-tissue swelling, and jaw prognathism, acromegaly is associated with a range of physical symptoms and significant comorbidities, including osteoarthritis, diabetes mellitus (DM), hypertension (HT), and respiratory and cardiac dysfunction [7]. The initial diagnosis of acromegaly relies on a high index of clinical suspicion and biochemical assessment, including elevated serum IGF-1 levels and failure of GH suppression during an oral glucose tolerance test (OGTT), with confirmation by pituitary magnetic resonance imaging (MRI) [8].

The management of acromegaly and its associated comorbidities is complex and necessitates a comprehensive, multidisciplinary approach [9]. Surgery is typically the first-line treatment of choice for patients with acromegaly. Patients who are not cured following surgery are generally treated with medical therapy, including somatostatin analogs (SSAs), dopamine agonists, and GH receptor antagonists. Radiation therapy is typically reserved for those who remain uncontrolled despite surgery and medical treatment [10].

This study aims to examine the demographic, clinical, and hormonal profiles; comorbidities; surgical pathology; treatment patterns; and long-term clinical outcomes of patients diagnosed with and followed at our center for acromegaly over a 15-year period from 2010 to 2024.

## 2. Materials and methods

### 2.1. Study design and patients

We retrospectively studied 247 acromegalic patients (106 males and 141 females) who were diagnosed, treated, and followed in our department from 2010 to 2024. Only patients with a minimum follow-up duration of 12 months after diagnosis were included. Exclusion criteria were age under 18 at diagnosis and incomplete laboratory or medical data in the follow-up records.

We reviewed the patients’ demographics, age at diagnosis, follow-up duration, and comorbidities. Additional data included biochemical laboratory results such as serum GH and IGF-1 levels adjusted for age and gender, GH response to OGTT, presence of pituitary hormone deficiency, tumor size and characteristics on MRI, pathology findings including pituitary hormone costaining, treatment modalities (preoperative and postoperative medical therapy, surgery, and radiation), and patients’ responses to these treatments.

The diagnosis of acromegaly was established according to the clinical guidelines prevailing at the time of patient presentation. Patients evaluated after 2014 were diagnosed in accordance with the Endocrine Society 2014 Clinical Practice Guideline [11], whereas those diagnosed prior to 2014 were assessed according to the internationally accepted criteria in use at the time. In all cases, the diagnosis was based on elevated age-adjusted serum IGF-1 levels (>1.3 times the upper limit of normal), compatible clinical features, and the presence of a pituitary adenoma on MRI, with biochemical confirmation as appropriate. For the present study, all diagnoses were retrospectively reviewed and confirmed to fulfill the diagnostic components of the 2024 Acromegaly Consensus Criteria [12], particularly with respect to biochemical confirmation and imaging findings.

### 2.2. Biochemical measurements and remission criteria

The GH and IGF-1 concentrations were measured by using chemiluminescence on an IMMULITE 2000 Xpi (Siemens Healthcare Diagnostics Inc.). Serum IGF-1 levels were compared with the age- and sex-adjusted normal reference values provided in the manufacturer’s instructions for use. During the study period, different assay platforms were used for GH and IGF-1 measurements. Hormonal values were interpreted according to assay-specific reference ranges and cut-off values in effect at the time of testing.

In our study, biochemical remission was defined as a normal age- and sex-adjusted IGF-1 level and either a random GH level < 1 ng/mL or a GH level < 1 ng/mL after an oral glucose load at the last follow-up assessment, regardless of residual tumor presence. Since the GH assay platforms used during the study period were not ultrasensitive, a GH nadir cut-off of <1 ng/mL following OGTT was applied in accordance with the Endocrine Society 2014 Clinical Practice Guideline [11]. In cases with discordant GH and IGF-1 levels, remission status was determined solely by normalized IGF-1 levels. Patients who achieved biochemical remission following surgery without the need for additional medical or radiotherapeutic interventions were classified as having a postoperative cure [12]. Biochemical remission was assessed at the most recent follow-up visit using the most recent hormonal measurements available. The first postoperative biochemical evaluation determines postoperative cure, typically performed 3–6 months after surgery.

### 2.3. Radiological imaging

MRI reports were reviewed both at the time of diagnosis and after surgical resection of the pituitary tumor. Tumor dimensions—including length (anteroposterior), width (transverse), and height (craniocaudal)—and Knosp grades were extracted from radiology reports and imaging data. The Knosp–Steiner classification was assessed based on coronal T1-weighted contrasted imaging. The largest measured dimension was recorded as tumor size, and adenomas with a maximum diameter ≥10 mm were classified as macroadenomas.

### 2.4. Surgical intervention

Surgical approaches included the endoscopic transnasal transsphenoidal approach and, in selected cases, craniotomy. The objective of surgery in all patients was complete resection of all visible tumor tissue or maximal debulking in cases of large, invasive adenomas.

### 2.5. Pathology evaluation

Immunohistochemical staining for pituitary hormones was routinely performed on surgical specimens of pituitary adenomas. Due to the retrospective nature of the study and variability in pathological reporting practices over the study period, GH immunostaining results were unavailable for all patients. During data collection, prolactin (PRL) coexpression was assessed based on findings reported in the pathology reports.

The patient selection process and formation of analytical subgroups are summarized in Figure.

## 3. Statistical analysis

The analyses were performed using SPSS 25.0 (IBM Corporation, NY, USA). The normality of the data was assessed using the Kolmogorov–Smirnov and Shapiro–Wilk tests, skewness–kurtosis, and graphical methods (histogram, Q–Q plot, stem-and-leaf plot, boxplot). For data with normal distribution, mean ± standard deviation was used, while median (interquartile range) was used for nonnormally distributed data. Categorical variables were presented as counts and percentages (n, %). For comparing continuous variables in dependent groups, the Student’s t-test was applied to those that followed a normal distribution, while the Mann–Whitney U test was used for those that did not. A p-value of < 0.05 was considered statistically significant.

## 4. Results

### 4.1. Baseline characteristics of patients

Table 1 summarizes the demographic, clinical, and comorbidity data of 247 patients [141 women (57.1%), 106 men (42.9%)] diagnosed with acromegaly. The mean age at the time of study was 53.3 ± 13.5 years, and the mean age at diagnosis was 43.4 ± 12.5 years. The median follow-up duration was 8 years (IQR: 3–14). At the time of diagnosis, the most frequent pituitary hormone deficiency was secondary adrenal insufficiency (hypocortisolism), present in 17.8% of patients, followed by hypogonadism in 12.5% and central hypothyroidism in 5.2%. Panhypopituitarism was rare, identified in only 2% of the cohort. Hyperprolactinemia was observed in 26.7% of cases.

Among 145 patients with available body mass index (BMI) data, the median BMI was 29.7 kg/m^2^ (IQR: 27.0–34.2), with 48.3% classified as obese (BMI > 30 kg/m^2^) and 43.4% as overweight (BMI 25–30 kg/m^2^). When stratified, 29% had class 1 obesity, 12.4% had class 2 obesity, and 6.9% had class 3 obesity. The most common comorbidities were HT (42.1%), DM (30.8%), and obstructive sleep apnea syndrome (10.1%). Coronary artery disease or congestive heart failure was present in 6.9% of patients.

Categorical data are presented as numbers and percentages, nonparametric data with a normal distribution are expressed as mean ± standard deviation (SD), while nonnormally distributed data are presented as medians (IQR 25–75).

### 4.2. Biochemical profile

Table 2 summarizes the hormonal profiles of patients with acromegaly at diagnosis and during follow-up. At the time of diagnosis, the median serum GH level was 8.5 mcg/L (IQR: 4.0–16.9), and the median IGF-1 level was 708.0 ng/mL (IQR: 546.0–929.5). The median nadir GH level 2 h after an OGTT was 4.7 mcg/L (IQR: 2.7–11.9). At 3 months postoperatively, there was a substantial reduction in hormone levels, with a median GH of 1.1 mcg/L (IQR: 0.42–2.96) and IGF-1 of 249.5 ng/mL (IQR: 179.0–394.0). At the most recent follow-up, median GH and IGF-1 levels further decreased to 0.90 mcg/L (IQR: 0.50–2.00) and 192.0 ng/mL (IQR: 127.5–261.5).

### 4.3. Imaging and histological characteristics of adenomas

Preoperative pituitary MRI was available for 193 of 247 patients to evaluate adenoma size and invasiveness. Among these, macroadenomas were present in 153 patients (79.3%), while microadenomas were detected in 40 patients (20.7%). The baseline median tumor dimensions were 14.0 mm anteroposterior (IQR: 9.5–20.0), 12.0 mm transverse (IQR: 8.2–17.0), and 12.0 mm craniocaudal (IQR: 9.0–17.0). Regarding the Knosp classification, 28.6% of tumors were grade 4, followed by 18.1% grade 3, 14.3% grade 2, 23.8% grade 1, and 15.2% grade 0. Immunohistochemical staining for GH was positive in 136 out of 143 tumors (95.1%), and 61 of these (42.7%) demonstrated costaining for PRL (Table 3).

### 4.4. Treatment and outcomes of patients

Therapeutic interventions and clinical outcomes are summarized in Table 4. A total of 247 patients were included in the study; however, treatment outcome data were available for 242 patients, as five patients were lost to follow-up prior to outcome assessment. Treatment choices were not significantly influenced by factors such as adenoma size, Knosp grade, or baseline GH/IGF-1 levels. Of the 242 patients, 15 (6.2%) received primary medical therapy without undergoing surgical intervention due to significant comorbidities or refusal of surgery. These patients are categorized as “only drug treatment” in Table 4. Initial treatments consisted of lanreotide monotherapy (46.7%), octreotide monotherapy (13.3%), or cabergoline monotherapy (13.3%), followed by octreotide and cabergoline combination therapy in 20.0% of cases; one patient received a combination of lanreotide and cabergoline. Biochemical remission was achieved in 85.8% of patients treated with lanreotide, whereas none were observed in patients receiving octreotide monotherapy. Most patients receiving combination therapy also did not achieve biochemical remission.

A total of 218 patients underwent surgical intervention; among them, 199 (91.3%) had transsphenoidal resection, while 19 (8.7%) underwent initial craniotomy. During the course of their disease, 23 patients required multiple surgical procedures, including three patients who underwent more than two craniotomies.

Medical therapy was administered to all patients with persistent disease following surgery (n = 86) and to the 15 patients who did not undergo surgery. Except for seven patients (8.1%) who received cabergoline monotherapy, all were treated with somatostatin analogs (SSAs), including octreotide or lanreotide. Lanreotide monotherapy was used in 25 patients (29.1%) and octreotide monotherapy in 22 patients (25.6%). Combination therapy was utilized in 32 patients, either sequentially or concurrently, SSAs with cabergoline in 29.1% of cases, and SSAs with pegvisomant (a GH receptor antagonist) in 8.1%.

Radiotherapy or radiosurgery, primarily the gamma knife, was used in 10.5% (26/247) of patients. Among these, two patients achieved remission without requiring any medical therapy. Biochemical remission was achieved in 13 patients (50.0%) following postoperative SSAs. Management is ongoing for patients who did not achieve remission.

Hormonal disturbances, including deficiency of arginine vasopressin as well as hypopituitarism requiring hormone replacement therapy, were observed in 22.8% of patients. Postoperatively, one or more anterior pituitary hormone deficiencies were found in 34 patients, with central hypothyroidism being the most common (52.9%). In four of these patients, hypopituitarism was attributed to radiotherapy rather than to the surgical intervention. Additionally, eight out of the 34 patients (23.5%) with hypopituitarism had undergone repeat surgical procedures. Among the 218 patients who underwent surgery, 86 (39.4%) achieved postoperative cure without adjuvant medical therapy or radiotherapy. Notably, 55 of these 86 patients (63.9%) had macroadenomas. Of the 242 patients with available clinical outcome data, 220 had complete biochemical follow-up data sufficient for remission assessment. At the most recent follow-up, 170 of 220 patients (77.3%) had achieved biochemical remission.

Table 5 summarizes the biochemical parameters, histopathological findings, and adenoma characteristics of patients based on biochemical remission status. Patients who achieved biochemical remission were significantly older than those who did not (p = 0.003). HT and DM were more prevalent among patients in the remission group (both p < 0.05). Patients who achieved biochemical remission had significantly lower GH levels (p = 0.022) and 2-h nadir GH levels after OGTT (p = 0.019) at diagnosis, as well as lower GH (p < 0.001) and IGF-1 levels (p < 0.001) at the most recent follow-up. Regarding adenoma size, patients who did not achieve biochemical remission had significantly larger anteroposterior (p = 0.005) and transverse (p = 0.011) diameters. Positive GH immunostaining was more common in patients who achieved remission (p = 0.017), while those who did not achieve remission were more likely to have higher Knosp grades (3–4) (p = 0.002).

## 5. Discussion

In this retrospective study of 247 acromegalic patients managed at a tertiary endocrine center over a 15-year period, we analyzed the demographic characteristics, clinical and biochemical profiles, treatment approaches, and long-term outcomes.

### 5.1. Patient demographics, clinical features, and adenoma characteristics

The demographic characteristics of our cohort are consistent with previously published data, demonstrating a female predominance (57.1%) and a mean age at diagnosis of 43.4 years. These findings align with recent epidemiological studies reporting mean ages at diagnosis ranging from 40 to 50 years [13]. The median follow-up period of 8 years in our study provides a robust framework for evaluating long-term treatment outcomes and efficacy.

Acromegaly is associated with numerous systemic complications resulting from chronically elevated levels of GH and IGF-1, including cardiovascular and respiratory diseases, metabolic disorders such as glucose and lipid abnormalities, HT, and osteoarthropathy. These are the main clinical conditions responsible for the increase in mortality associated with the disease [14]. In our cohort, the most frequently observed comorbid conditions were obesity (48.3%), HT (42.1%), and DM (30.8%), consistent with previous studies [15,16]. The increased prevalence of these metabolic comorbidities is attributed to the insulin-antagonistic effects of excess GH and IGF-1, leading to insulin resistance and impaired glucose metabolism [17]. Hyperprolactinemia was observed in 26.7% of our patients at the time of diagnosis. These findings are consistent with earlier reports indicating that up to one-third of somatotroph adenomas cosecrete PRL, potentially contributing to additional clinical manifestations such as hypogonadotropic hypogonadism and galactorrhea [18,19]. Hyperprolactinemia may be attributed to either cosecretion of PRL by the tumor or the stalk effect, both of which are known to disrupt dopaminergic inhibition of PRL secretion. In our cohort, immunohistochemical analysis demonstrated PRL costaining in 42.7% of adenoma specimens, suggesting a substantial prevalence of mammosomatotroph adenomas. This finding is consistent with the recent study by Lawrence et al. (Acromegaly: a clinical perspective, 2020), which reported PRL costaining in 41.9% of cases. In our cohort, the most frequently observed pituitary hormone deficiency was hypocortisolism (17.8%), aligning with previous studies that highlight adrenocorticotropic hormone deficiency as a common finding in patients with macroadenomas due to tumor-related compression of corticotroph cells [15].

Our findings demonstrate that the majority of patients presented with macroadenomas (79.3%). The predominance of macroadenomas underscores the delayed diagnosis that often characterizes acromegaly, as symptoms develop insidiously and may be overlooked for years.

A recent large multicenter study involving patients with biochemically confirmed acromegaly reported that 56% of GH-secreting pituitary tumors exhibited cavernous sinus invasion, as identified by imaging or intraoperative assessment [20]. Other contemporary surgical series that utilize direct intraoperative observation and histopathological evaluation have reported cavernous sinus invasion rates in somatotroph adenomas ranging from approximately 37% to over 89%, depending on the detection methods and criteria used [21,22]. The majority of patients present with macroadenomas, which are more likely to invade the cavernous sinus [20]. The high proportion of invasive adenomas in our cohort (Knosp grades 3–4 in 46.7%) reflects the locally aggressive nature of many GH-secreting tumors, a finding consistent with previous studies reporting cavernous sinus invasion.

### 5.2. Treatment outcomes

Despite the advanced stage of disease at presentation, 77.3% of patients ultimately achieved biochemical remission following a combination of surgical, medical, and radiotherapeutic interventions.

Surgical resection of the adenoma is considered the first-line treatment option for most patients with acromegaly. The endoscopic transsphenoidal approach has been widely adopted as the primary modality for pituitary adenoma resection since the 1990s, offering improved clinical outcomes and a lower complication rate compared with craniotomy [23]. In the current analysis, transsphenoidal surgery was the first treatment option for many of our patients (91.3%). Although preoperative medical treatment can reduce adenoma size and improve surgical cure rates, routine use of medical therapy for this purpose is not recommended, as evidence for a benefit on postoperative outcomes remains unclear [24,25]. Primary medical therapy is typically reserved for patients at high surgical risk, such as those with serious cardiomyopathy and respiratory disease, or those who refuse surgery [9]. In our cohort, 15 patients (6.2%) received only drug treatment for the same reasons previously outlined, with the majority of them (46.7%) being treated with lanreotide. For patients with persistent disease after surgery, repeat surgeries by an experienced surgeon may be useful when the tumor is accessible. In a recent study, repeat surgery was performed in 14 patients who had failed initial surgery, and 57% achieved biochemical control [26]. In our cohort, 23 patients who have failed initial surgery required multiple surgeries during the course of their disease, with three patients undergoing more than two craniotomies. Among these 23 patients, 39.1% achieved biochemical remission with adjuvant therapy.

IGF-1 levels measured 3 months after surgery serve as a valid marker of surgical cure [9]. In our cohort, postoperative 3-month GH and IGF-1 levels were 1.1 mcg/L and 249.5 ng/mL, respectively. Among the 218 patients who underwent surgery, 86 (39.4%) achieved postoperative cure without the need for adjuvant therapy, which aligns with published rates of 25,3–84,7% depending on case selection, tumor size, invasiveness, and surgical expertise [27]. Notably, 63.9% of surgically cured patients had macroadenomas, suggesting that tumor size alone does not preclude successful surgical outcomes.

For patients with persistent disease after surgery, medical therapy played a pivotal role in achieving biochemical control. First-generation SSAs (octreotide and lanreotide) were the most commonly used agents in our cohort, which is consistent with current guidelines recommending SSAs as first-line medical therapy [24]. In our cohort, lanreotide monotherapy yielded the highest remission rates among pharmacological treatments. Cabergoline or pegvisomant was reserved for resistant or residual disease. These findings underscore the need for individualized treatment strategies and reinforce the utility of multimodal therapy in achieving disease control. Combination therapy was utilized in a significant proportion of our patients, with SSAs plus cabergoline being the most common combination (29.1% of medically treated patients). This approach is supported by recent data showing that adding cabergoline to SSA therapy can provide additional IGF-1 normalization in 42% of partial responders [28]. The use of pegvisomant in combination with SSAs was limited to 8.1% of our medically treated patients, despite its established efficacy in normalizing IGF-1 levels in up to 96% of patients [29]. This relatively low utilization may reflect limited access to medication, cost considerations, or concerns about hepatotoxicity and tumor growth.

Radiotherapy or radiosurgery was employed in 10.5% of our patients, primarily as a third-line approach for cases resistant to surgical and medical interventions. 50% of these patients achieved biochemical remission following radiotherapy and adjuvant medical therapy. A large-scale international multicenter retrospective cohort study by Ding et al. (2019) demonstrated that serum IGF-1 normalization rates were 51%, 69%, and 74% at 5, 10, and 15 years posttreatment, respectively, following SSAs [30]. This temporal pattern underscores the importance of long-term follow-up in assessing the effectiveness of radiotherapy, as reflected by our cohort’s median follow-up duration of 8 years. Notably, two patients achieved biochemical remission without concurrent medical therapy. For those who have not yet achieved remission, ongoing therapeutic management remains essential, given the delayed onset and the typically protracted timeline required for radiotherapy to exert its full therapeutic effect. Among patients who received radiotherapy, data on hypopituitarism were incomplete. Of the 26 patients who underwent radiotherapy, only one had not undergone surgery. Hypopituitarism was detected in six patients; in two of them, it was newly diagnosed following radiotherapy. However, the exact timing of RT administration was not available. Therefore, the incidence of radiotherapy-related hypopituitarism may be underestimated and could increase with longer follow-up.

Reported rates of new anterior pituitary deficits following surgical intervention for acromegaly vary widely in the literature, ranging from 4% to 39% [31]. A systematic review and metaanalysis evaluating 92 studies encompassing 6988 patients documented a weighted incidence rate of 12.79% for postsurgical hypopituitarism [32]. In the current analysis, postoperative anterior pituitary hormone deficiencies were observed in 22.8% of patients, with central hypothyroidism being the most common (52.9%). Notably, the prevalence of postoperative hypopituitarism has declined over time in the literature, from 41% to 23%. This trend likely reflects advancements in surgical techniques, the broader use of pharmacologic therapies, and a reduced reliance on radiation therapy in the management of acromegaly [31].

Our analysis identified several factors associated with biochemical remission. Patients who achieved remission were significantly older at diagnosis (p = 0.003) and had lower baseline GH levels (p = 0.022) and lower nadir GH levels following OGTT (p = 0.019). Previous studies have also explored age as a potential predictor of biochemical remission in acromegaly, showing that older patients tend to have lower baseline GH secretion, which may contribute to improved treatment outcomes compared with younger individuals [33]. The inverse relationship between baseline GH levels and treatment success likely reflects less aggressive disease biology and better responsiveness to therapy.

Interestingly, our analysis revealed that patients who achieved biochemical remission had significantly higher rates of HT and DM compared with those who did not. This finding may partly be explained by age, as patients in the remission group were significantly older at diagnosis, and both HT and diabetes are more prevalent in older populations. Differences in disease duration or tumor characteristics may also have contributed to this observation. While this may initially seem paradoxical, patients with these comorbidities may have had more frequent healthcare interactions, leading to earlier diagnosis, closer monitoring, and more aggressive or sustained treatment. Alternatively, this association could reflect a selection bias, where patients with more overt metabolic complications were prioritized for, or were more adherent to, therapy. Further prospective studies are warranted to determine whether these comorbid conditions influence treatment responsiveness or merely reflect better access to care.

Consistent with previous studies, macroadenomas were the predominant tumor subtype in our cohort, accounting for nearly 80% of cases. High Knosp grades (3–4), which are indicative of cavernous sinus invasion, were significantly more common in patients who did not achieve biochemical remission, underscoring the prognostic value of tumor invasiveness in surgical outcomes. Patients with lower Knosp grades (0–2) demonstrated higher rates of biochemical remission, aligning with previous studies that emphasize the impact of tumor extension on surgical success [27]. Tumor dimensions, especially the anteroposterior and transverse diameters, were significantly larger in the nonremission group, underscoring the prognostic importance of tumor size in predicting treatment outcomes.

Additionally, positive GH immunostaining in tumor histopathology was associated with a higher likelihood of achieving biochemical control, underscoring the potential prognostic value of immunohistochemical markers. In contrast, weaker staining may indicate a less differentiated tumor phenotype, possibly linked to more aggressive or unpredictable clinical behavior. However, such findings should be interpreted cautiously and within the broader context of accompanying pathological and clinical characteristics.

Our study benefits from a relatively large sample size and an extended follow-up period, enabling a robust assessment of treatment outcomes in patients with acromegaly. However, several limitations should be acknowledged. As a single-center retrospective analysis, it is inherently subject to selection and information biases, and its findings may not be fully generalizable to other populations or healthcare settings. Changes in diagnostic criteria, therapeutic approaches, radiotherapy techniques, and dosing regimens over the 15-year study period may have introduced variability, complicating the interpretation of outcomes. In addition, surgeon experience and potential learning-curve effects were not specifically evaluated and may have influenced postoperative outcomes. Although biochemical remission was defined according to contemporary guidelines and assay-specific reference ranges were applied, changes in laboratory platforms and measurement methods over time may have introduced minor variability and limited comparability with other cohorts. Furthermore, while the median follow-up duration of 8 years is substantial, the delayed therapeutic effects of radiotherapy may necessitate even longer observation periods for a complete evaluation. Mortality data were not consistently available in this retrospective cohort; therefore, survival analysis could not be performed, as mortality was not a predefined endpoint of the study. Finally, incomplete histopathological and imaging data in a subset of patients—particularly regarding Knosp classification and immunohistochemical staining—may have constrained the depth of certain analyses.

In conclusion, our 15-year retrospective analysis of 247 patients with acromegaly offers valuable insights into the clinical presentation, management strategies, and long-term outcomes of the disease in a real-world setting. The findings underscore the critical importance of early diagnosis and individualized, multimodal treatment approaches. Favorable outcomes were associated with older age at diagnosis, lower baseline GH levels, positive GH immunostaining, smaller tumor dimensions, and lower Knosp grades (0–2), highlighting the prognostic significance of both biochemical and anatomical factors.

Early diagnosis, successful surgical removal of the tumor, and personalized treatment plans—tailored to the tumor’s invasiveness, hormone secretion patterns, and the patient’s other health conditions—are key to achieving effective disease control in acromegaly. Future prospective studies are warranted to refine prognostic markers and establish standardized treatment algorithms aimed at improving long-term outcomes in this patient population.

## Figures and Tables

**Figure:**
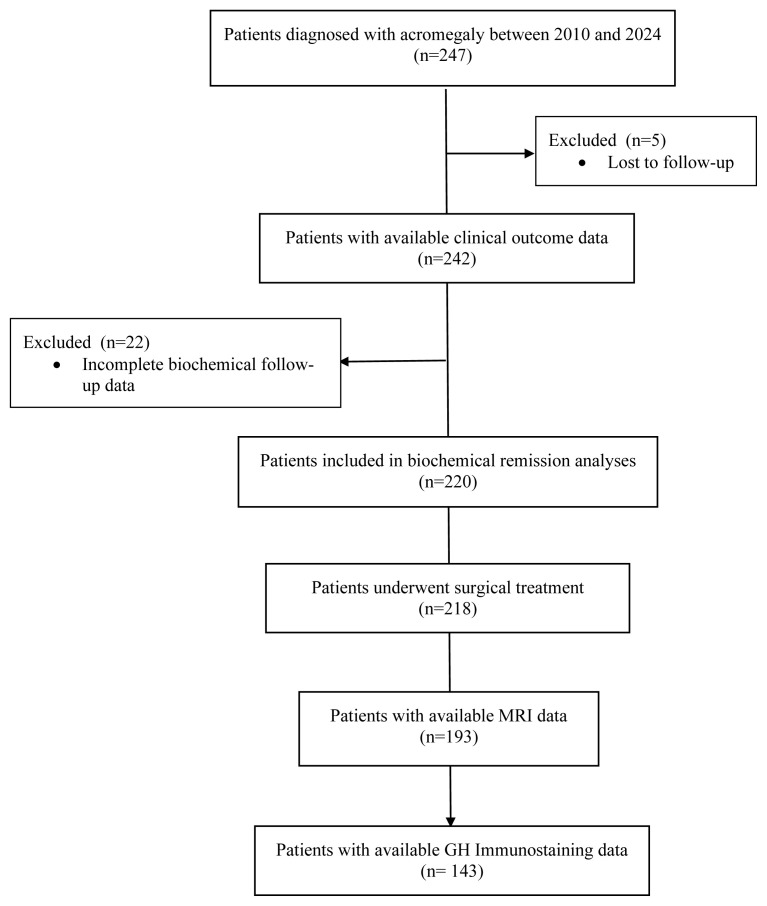
Flow diagram of patient selection and analytical cohotrs.

**Table 1:** Baseline demographic, clinical features, and comorbidities of patients.

Total n = 247	n (%) or Mean ± SD or median (IQR 25–75)
Sex	
Female	141 (57.1)
Male	106 (42.9)
Age, years	53.3 ± 13.5
Age at diagnosis, years	43.4 ± 12.5
Follow-up duration, years	8.0 (3.0–14.0)
Hypopituitarism	
Hypocortisolism	44 (17.8)
Hypothyroidism	13 (5.2)
Hypogonadism	31 (12.5)
Panhypopituitarism	5 (2.0)
Hyperprolactinemia	66 (26.7)
BMI, kg/m^2^ (n = 145)	29.7 (27.0–34.2)
Obesity (BMI > 30 kg/m^2^)	70 (48.3)
Overweight (BMI: 25–30 kg/m^2^)	63 (43.4)
Class 1 obesity (BMI: 30–35 kg/m^2^)	42 (29.0)
Class 2 obesity (BMI: 35–40 kg/m^2^)	18 (12.4)
Class 3 obesity (BMI: > 40 kg/m^2^)	10 (6.9)
Diabetes Mellitus	76 (30.8)
Hypertension	104 (42.1)
OSAS	25 (10.1)
Coronary artery disease/congestive heart failure	17 (6.9)

BMI: Body mass index, OSAS: Obstructive sleep apnea syndrome

**Table 2:** Laboratory findings of patients.

	Median (IQR 25–75)
IGF-1 level at diagnosis, ng/ml	708.0 (546.0–929.5)
GH level at diagnosis, mcg/L	8.5 (4.0–16.9)
2-h post-OGTT GH level at diagnosis, mcg/L	4.7 (2.7–11.9)
Postoperative 3 months IGF-1 level, ng/ml	249.5 (179.0–394.0)
Postoperative 3 months GH level, mcg/L	1.1 (0.42–2.96)
Most recent IGF-1 level, ng/ml	192.0 (127.5–261.5)
Most recent GH level, mcg/L	0.90 (0.50–2.00)

IGF-1: insulin-like growth factor 1, GH: growth hormone, OGTT: oral glucose tolerance test

**Table 3:** Adenoma features on imaging and histopathology.

Total n = 193	n (%) or median (IQR 25–75)
Macroadenoma	153 (79.3)
Microadenoma	40 (20.7)
Tumor size	
Tumor length/anteroposterior, mm	14.0 (9.5–20.0)
Tumor width/transverse, mm	12.0 (8.2–17.0)
Tumor height/craniocaudal, mm	12.0 (9.0–17.0)
Knosp grade (n = 105)	
0	16 (15.2)
1	25 (23.8)
2	15 (14.3)
3	19 (18.1)
4	30 (28.6)
GH staining (n = 143)	136 (95.1)
PRL costaining (n = 143)	61 (42.7)

GH: growth hormone, PRL: prolactin

**Table 4:** Treatment and outcomes of patients.

Total n = 247	n	n (%)
Only drug treatment	242	15 (6.2)
• Octreotide	15	2 (13.3)
• Lanreotide	15	7 (46.7)
• Cabergoline	15	2 (13.3)
• Octreotide + cabergoline	15	3 (20)
• Lanreotide + cabergoline	15	1 (6.7)
Transsphenoidal surgery	218	199 (91.3)
Transcranial surgery	218	19 (8.7)
Multiple surgery	218	23 (9.3)
Medical treatment after surgery		
• Octreotide	86	22 (25.6)
• Lanreotide	86	25 (29.1)
• Cabergoline	86	7 (8.1)
• Octreotide + cabergoline	86	12 (14.0)
• Lanreotide + cabergoline	86	13 (15.1)
• Octreotide + pegvisomant	86	5 (2.3)
• Lanreotide + pegvisomant	86	2 (5.8)
Radiotherapy	247	26 (10.5)
Hypopituitarism	149	34 (22.8)
• Central hypocortisolism	34	10 (29.4)
• Central hypothyroidism	34	18 (52.9)
• Central hypogonadism	34	14 (41.1)
• Central DI	34	13 (38.2)
• Panhypopituitarism	34	2 (5.8)
Postoperative cure	218	86 (39.4)
Respond to medication and/or radiotherapy	220	170 (77.3)

DI: Diabetes insipidus

**Table 5:** Biochemical parameters, histopathological findings, and adenoma characteristics of patients based on biochemical remission status.

	Biochemical remission (n = 170)	Biochemical nonremission (n = 50)	P-value*
n (%) or Mean ± SD or Median [IQR 25–75]	n (%) or Mean ± SD or Median [IQR 25–75]		
Female gender	98 (57.6)	25 (50.0)	0.338
Age, years	56.0 ± 12.8	49.8 ± 12.9	**0.003**
BMI, kg/m^2^	29.5 (27.0–31.3)	29.5 (27.0–31.3)	0.562
Diabetes mellitus	65 (38.2)	9 (18.0)	**0.008**
Hypertension	82 (48.2)	16 (32)	**0.042**
OSAS	18 (15.3) (n = 118)	4 (9.5) (n = 42)	0.354
Coronary artery disease/congestive heart failure	16 (9.4)	1 (2)	0.084
IGF-1 level at diagnosis, ng/ml	655.0 (534.0–882.0)	793.0 (550.0–1020.0)	0.193
GH level at diagnosis, mcg/L	7.8 (3.9–13.6)	14.1 (4.6–24.2)	**0.022**
2-h post-OGTT GH level at diagnosis, mcg/L	4.2 (2.6–10.9)	8.9 (3.3–17.2)	**0.019**
Most recent IGF-1 level, ng/mL	168.5 (118.0–212.0)	325.0 (254.5–542.5)	**0.000**
Most recent GH level, mcg/L	0.8 (0.3–1.3)	3.3 (1.9–7.1)	**0.000**
Macroadenoma	98 (76.6) (n = 128)	36 (87.8) (n = 41)	0.122
Tumor length/anteroposterior, mm	13.0 (9.0–18.0)	18.0 (11.0–25.5)	**0.005**
Tumor width/transverse, mm	11.0 (8.0–16.0)	15.5 (10.0–19.5)	**0.011**
Tumor height/craniocaudal, mm	12.0 (9.0–16.0)	17.0 (9.0–22.0)	0.059
GH staining	97 (97.0) (n = 100)	23 (85.2) (n = 27)	0.017
Prolactin costaining	42 (42.0) (n = 100)	10 (37.0) (n = 27)	0.642
Knosp grade 0 – 1–2	39 (60.9) (n = 64)	5 (22.7) (n = 22)	**0.002**
Knosp grade 3–4	25 (39.1) (n = 64)	17 (77.3) (n = 22)	

* Statistically significant data are highlighted in bold
